# Engaging Knowledge Users with Mental Health Experience in a Mixed-Methods Systematic Review of Post-secondary Students with Psychosis: Reflections and Lessons Learned from a Master’s Thesis

**DOI:** 10.34172/ijhpm.2020.138

**Published:** 2020-08-01

**Authors:** Victoria Sanderson, Amanda Vandyk, Jean Daniel Jacob, Ian D. Graham

**Affiliations:** ^1^School of Nursing, Faculty of Health Sciences, University of Ottawa, Ottawa, ON, Canada.; ^2^School of Epidemiology and Public Health, University of Ottawa, Ottawa, ON, Canada.

**Keywords:** Integrated Knowledge Translation, Systematic Review, Knowledge User, Psychosis

## Abstract

Engaging knowledge users (KUs) as research team members throughout the research process helps generate relevant knowledge and may improve uptake of research results. The purpose of this article is to describe how an integrated knowledge translation (iKT) approach was embedded within a master’s thesis project comprising a mixed-methods systematic review. KUs were engaged in four distinct phases of the systematic review process, including (1) proposal development; (2) development of the research question and approach; (3) creation of an advisory panel; and (4) an end of study meeting to interpret findings and plan dissemination of findings. The extent of each KU’s engagement on the research team fluctuated during the study. Challenges included maintaining the same KUs throughout the project and maintaining the scope of the project to align with a master’s thesis. Our suggestions for optimizing graduate student iKT projects include having regular team meetings and periodically checking in with team members to encourage reflection on overall engagement and progress of the project. Overall, KUs helped create a research project designed to address their needs and provided input on how results might translate into implications for clinical practice, education, academic policy, and future research within their respective contexts.

## Background

Health-related research should inform the delivery of health services, yet translation of research findings into meaningful practice change is difficult.^1^ Within the Canadian context, integrated knowledge translation (iKT) is an approach to conducting research that helps increase research relevance, applicability, feasibility, and impact.^1^ iKT is “…a model of collaborative research, where researchers work with [knowledge users, KUs] who identify a problem and have the authority to implement the research recommendations”(p. 299).^2^ iKT involves KUs – as equal partners – who play an integral role in the research process.^3^ This approach to conducting research has similarities with participatory methods (eg, participatory action research, community-based participatory research),^4^ including the desire to co-create research questions and advance knowledge to improve current conditions.^5^ Participatory research emphasizes community-driven solutions with a focus on social justice,^5,6^ whereas iKT emphasizes research-based solutions.^4^ Co-creation emerged from several fields simultaneously, including business study and design science, and has similarities with iKT, including working alongside end-users at various stages in the research process to increase research impact.^6^

The term ‘knowledge user’ is used within iKT research to describe individuals who are likely to use research results to make informed decisions,^3^ but similar terms are also used (eg, stakeholders, decision-makers) across international literature on the subject.^6^ Systematic reviews, a type of research study amenable to using an iKT approach, provide high quality evidence,^7^ inform clinical practice guidelines, and serve as a framework for clinical decisions.^8^ Graham and colleagues report that using an iKT approach when conducting systematic reviews can lead to better uptake of research results, because the people for whom the results are pertinent are engaged throughout the process, ensuring the research is targeted to their needs.^1^ This approach to research proposes collaboration between researchers and KUs, in an attempt to achieve societal impact, where research results inform KU decisions (including clinicians, managers, policy-makers, patients, etc).^1^

iKT-driven systematic reviews are gaining popularity and are being conducted in the field of healthcare. For example, Hemens and colleagues conducted a systematic review in partnership with senior hospital managers and clinical leaders to assess effective choices for computerized clinical decision support systems.^9^ Pollock and colleagues involved stakeholders (patients, carers, and physiotherapists) in the update of a Cochrane systematic review relating to physiotherapy after stroke, and researchers explored the impact of involving stakeholders in their study.^10^ Hyde and colleagues involved a patient ‘research user group’ as part of the research team in conducting a systematic review on shared decision-making in prescribing analgesia in a primary care consultations.^11^

The purpose of this article is to describe how iKT was embedded within a mixed-methods systematic review.^12^ The mixed-methods systematic review was a master’s thesis project. We outline various KU engagement activities employed throughout the systematic review process, reflect on lessons learned, and provide suggestions for students conducting iKT-informed research projects. The paper is divided in 3 sections: (1) a description of the iKT process; (2) the results of the iKT engagement as they map onto these steps; and (3) a methodological discussion.

## Methods

**Figure 1:**
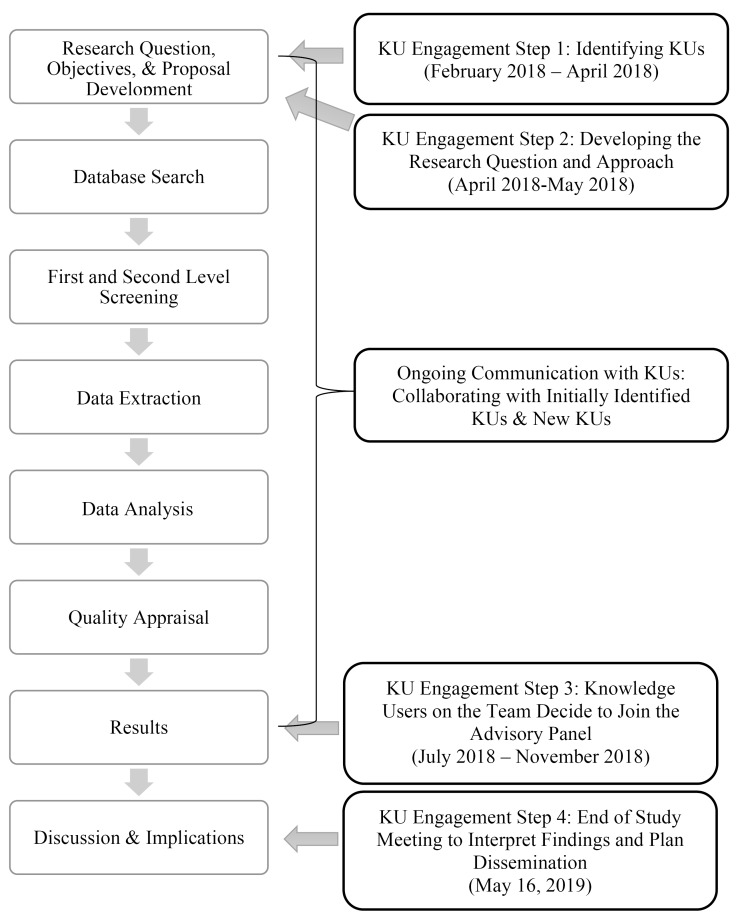
Knowledge User Engagement Steps Throughout the Systematic Review Process. Abbreviation: KU, knowledge user.

The research team included a registered nurse with mental health experience (the primary investigator [PI], pursuing a master’s of nursing degree), the thesis committee (consisting of 3 university professors), 2 peers pursuing graduate nursing degrees (who contributed to citation screening, data extraction, and quality appraisal), and over the course of the study, a total of (n= 11) KUs were involved. This project was constrained by the master’s-degree scope and timeline of approximately 12 months. The iKT project was informed by the Canadian Institutes of Health Research (CIHR) Guide to Knowledge Translation Planning^3^ and the CIHR guide to collaborating with KUs.^13^ KU engagement for the systematic review was also guided by the work of Keown and colleagues,^14^ and Guise and colleagues.^15^ Keown and colleagues outlined opportunities to involve KUs (referred to as ‘stakeholders’ in the article) during the conduct of a systematic review, such as consulting during research proposal development, discussing preliminary results, and participating in dissemination activities.^14^ Guise and colleagues provided an overview of KU engagement practices based on a literature review and interviews with leaders from research, policy, and evidence-based practice organizations.^15^ We outline KU engagement steps in the following section (see Figure 1).

###  Step 1: Identifying the Knowledge Users

The PI sought to pursue a research project investigating psychosis in post-secondary (PS) students and wanted to use an iKT approach to target potential information needs of KUs. Using the CIHR Guide to Researcher and KU Collaboration in Health Research, potential KUs for this project could be anybody who may benefit or be otherwise affected by research results.^13^ This could include PS students with psychosis, educators with mental health experience, clinicians who provide care to PS students with psychosis, policy advisors within PS institutions who may influence policy on mental health, and persons who work in community organizations or groups involved in improving mental healthcare for PS students.

To identify potential KUs, the PI examined the context and environment in which the research was taking place.^13^ This included perusing websites of potential partner organizations, networking through personal connections, attending community events, and informal meetings with potential KUs.^13^ As a first step, the PI searched for contact information on local PS institution websites (to identify clinicians, educators, and policy advisors who work within PS institutions) and local hospital websites (to identify clinicians who work within psychiatric services). As a second step, the PI reached out to personal contacts.

Once identified, the PI connected with each potential KU individually and provided introductions and a brief overview of the research area of interest. An informal meeting and understanding of the project and roles of KUs is likely sufficient for smaller projects, such as a master’s thesis.^13^ At this point, the PI explained that the research proposal was not yet formalized. The PI ensured that the potential KUs understood what iKT meant for this project and that at this stage, interest was being gauged, as well as relevance and feasibility of conducting research on psychosis in PS students.

 Consultation with the Ethics office at the University of Ottawa confirmed that while our research was exempt from ethics review, we were advised to obtain written documentation from each KU describing their role and understanding of how their involvement would be acknowledged.

###  Step 2: Developing the Research Question and Approach 

Prior to the second KU engagement step, the PI conducted a preliminary search of the available literature on psychosis in PS students to determine the types of studies and information available to meet the study objectives. Next, the PI re-connected with KUs identified in step one individually via email, telephone, or in-person, based on individual preference, to collaborate on research question(s) and approach. To reach consensus, after gathering ideas from each KU regarding research objectives and their foreseen roles as KUs, the PI combined KU feedback and emailed a summary of proposed research objectives and protocol, to each KU. Each KU replied via email to the proposed research objectives and protocol indicating whether they agreed with the outlined study objectives and protocol. As suggested by Parry and colleagues,^13^ the PI created and emailed an informal written partnership agreement which included a summary of proposed research objectives, protocol, and KU roles. Written partnership agreements are advantageous, as they provide clarity and future reference for team members.^13^

###  Ongoing Communication With Knowledge Users

The PI maintained ongoing communication with KUs. This included re-affirming the project’s objectives and circulating regular memos updating everyone on progress and preliminary results, as suggested by Parry and colleagues.^13^

###  Step 3: Knowledge Users on the Team Decide to Join the Advisory Panel 

 Once we completed the systematic review, up to and including the results, the PI formed an advisory panel of KUs involved throughout the duration of the study, to attend a meeting to provide input on results and dissemination activities. Prior to the meeting, the PI sent out an e-mail invitation to each KU involved throughout the duration of the study, with a summary of the project objectives, methods, and preliminary results. The invitation included goals of the end of study meeting (to discuss implications for clinical practice, education, academic policy, future research, and dissemination activities). The invitation included a letter outlining possible ways to acknowledge KU contributions to the project, and a meeting agenda. The PI encouraged discussion and questions prior to the meeting.

###  Step 4: End of Study Meeting to Interpret Findings and Plan Dissemination

An in-person meeting was held after systematic review results were generated and after the advisory panel was formed. It was held in a conference room at a PS institution. After introductions and an explanation of the meeting objectives, the PI presented a 20-minute summary of the systematic review (including objectives, methods, and a summary of results) and facilitated a discussion and feedback session about the implication of the study for the KUs. The PI retrieved written agreement from each KU on the advisory panel acknowledging how they wished to be acknowledged for their involvement in the study. Meeting minutes were documented in a Microsoft Word document. For all attendees, parking was reimbursed, and light snacks were provided, as suggested by Parry and colleagues.^13^ Following the meeting, the PI sent an email to each KU with meeting minutes and encouraged additional feedback via in-person meeting, telephone, or email.

## Results

###  Step 1: Identifying the Knowledge Users

 A total of 9 potential KUs were identified via institutional websites (n = 5) and personal connections (n = 4). The PI sent individual emails to the 9 potential KUs. After meeting with each person individually to discuss the direction of the research, 7 individuals agreed to join the research team. There was one registered nurse in a psychiatric outpatient setting, 2 medical doctors and one social worker in a PS institution, one nursing student interested in mental health, one student with lived-experience of psychosis, and one director of health and wellness services in a PS institution.

###  Step 2: Developing the Research Question and Approach 

 First, the PI presented a preliminary scan of the available literature on psychosis in PS students with proposed study objectives to KUs. Next, the PI gathered feedback via telephone and email from each KU on their ideas for proposed study objectives. The PI synthesized KU feedback and emailed the list of proposed study objectives to each KU. Finally, each KU emailed the PI indicating their agreement on the study objectives and direction of the project. Formalized study objectives included gathering knowledge to date on characteristics of PS students, prevalence of symptoms of psychosis among PS students, risk factors that may contribute to the development of symptoms of psychosis, interventions for PS students with symptoms of psychosis, and the experiences of PS students with symptoms of psychosis. Since objectives were broad and sought to appraise the literature to date on PS students with symptoms of psychosis, the PI suggested conducting a systematic review, which KUs agreed was the best method to address each objective.

###  Ongoing Communication With KU Team Members: Collaborating With Initially Identified KUs and New KUs

Mid-way through the project, after re-connecting with the 7 KUs, 4 KUs informed the PI they could no longer dedicate time to the project due to competing commitments, leaving a total of 3 KUs who remained involved in the project. At the same time, the PI attended a community event hosted by the Center for Innovation in Campus Mental Health, intended to encourage collaboration and partnership building between local campuses and community mental health organizations. The Center for Innovation in Campus Mental Health is a partnership project aimed at helping colleges and universities enhance their capacity to support student mental health.^16^ The PI utilized this forum as a chance to network and discuss the research project on PS students with psychosis, and coincidentally connected with 4 additional potential KUs. The PI met with each individual at a later date to discuss the research project, and each person expressed their desire to be involved and agreed to participate as a KU while the systematic review was in progress. KUs included an occupational therapist working for a first-episode psychosis program, a nursing professor of mental health at a PS institution, a mental health policy advisor at a PS institution, and a research scientist involved with improving youth mental health services and supports. Thus, a total of 7 KUs were members of the research team mid-way through the project.

###  Step 3: Knowledge Users on the Team Decide to Join the Advisory Panel 

**Figure 2:**
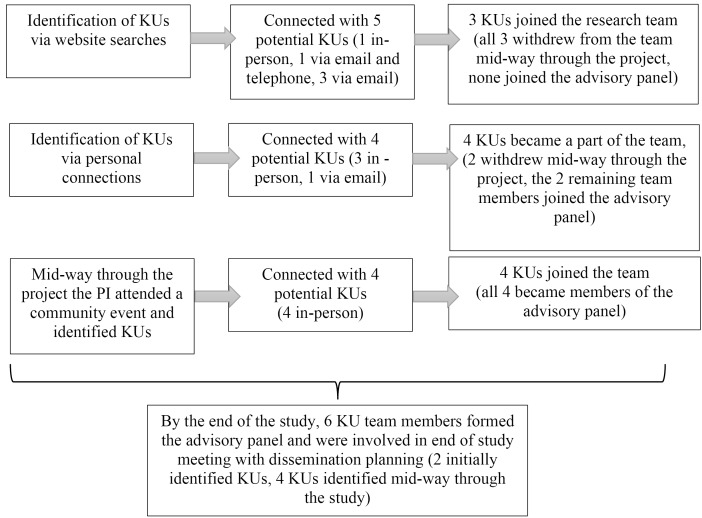
Knowledge User Engagement Throughout the Study. Abbreviations: KU, knowledge user; PI, primary investigator.

All KUs involved throughout the project, including those who declined involvement mid-way through the project, were invited to be a part of the advisory panel (n= 11). Five declined involvement in the advisory panel, providing the explanation of having competing job commitments, other research commitments, or life commitments. Two of these advisory panel members were KUs who joined the research team at the onset of study, while the remaining 4 KUs had joined the team mid-way through the study. Therefore, the final advisory panel consisted of 6 KUs, which is consistent with the ‘small group’ proposed by Keown and colleagues (p. 68).^14^ The composition of the advisory panel included an occupational therapist working for a first-episode psychosis program, a nursing professor of mental health at a PS institution, a mental health policy advisor at a PS institution, a PS student with lived experience of psychosis, a research scientist involved with improving youth mental health services and supports, and a nursing student interested in mental health. See Figure 2 for an illustration of KU engagement throughout the project.

###  Step 4: End of Study Meeting to Interpret Findings and Plan Dissemination

 The PI emailed a ‘doodle poll’ survey to each KU to reach consensus on a date and time of the meeting. Although all 6 KUs agreed on the same date and time, at the last minute 2 KUs could not attend. The PI, thesis committee members, one peer pursuing a graduate nursing degree, and 4 KUs attended the reaction and interpretation meeting. Together, we discussed implications for clinical practice, academic policy, education, future research, and strategies for dissemination of the findings through KU networks (and more broadly). The entire meeting lasted one and half hours and minutes were recorded in a Microsoft Word document. The PI organized the meeting minutes using narrative summaries according to each category addressed during the meeting. This included implications of results for clinical practice, academic policy, education, future research, and strategies for dissemination of findings. KUs provided additional feedback over email after the meeting minutes were sent out. Two KUs, who were unable to attend the end of study meeting, jointly provided additional in-person feedback to the PI at a later date.

## Discussion

###  Reflections on iKT Research Within the Scope of a Master’s Thesis Project: Lessons Learned

 Through this study, we came to appreciate the dynamic nature of an iKT-driven systematic review, as KU involvement ebbed and flowed over the course of the study. Here, we identify lessons learned and recommendations for future graduate student iKT projects.

First, we struggled to contain the scope of the project to fit within a master thesis. When developing the research objectives and protocol, the PI engaged with and met various KUs team members individually. Each KU proposed similar research questions however some questions were different, and the PI attempted to reconcile each research question into a systematic review large in scope with 5 distinct objectives. As with all research, priority setting is important for systematic review studies to identify the most important research gaps and health evidence needs.^17^ Moving forward with future iKT projects, we will more carefully consider the prioritization issue and use existing processes (eg, The Cochrane Collaboration Review Groups frameworks, approaches, or methods for prioritization in systematic reviews), which may help to guide preliminary KU conversations and narrow the focus.^18^ In this study, we reached consensus on study objectives by gathering individual feedback from KUs, compiled each idea into a project large in scope, and then received agreement from each KU individually. Ideally, if possible, having team meetings with all team members (either face-to-face or virtually) during the study proposal phase might facilitate reaching consensus on project priority objectives to make the study more manageable and smaller in scope for a master’s thesis.

 The second challenge was maintaining the same KU team members. The PI purposefully re-connected with each KU a minimum of every 2 months via e-mail or telephone, to foster on-going communication and discuss research progress. While 2 out of 7 original KU research team members stayed with the project to its completion, 5 out of 7 did not. To minimize or prevent KU turnover, the PI introduced new KUs into the team mid-way through the project.The KUs who joined the team mid-way through the project were interested and engaged, however they did not have the chance to provide input during the study proposal phase. While team member turnover is common to many research projects, as a graduate student, having to navigate and adapt to the realities of a changing research team was difficult. Specifically, having to replace KUs while the study was in progress was time consuming and threatened the timely completion of the study. Engagement strategies were limited to individual or small-group meetings, that were often via telephone, or via email, and having one team meeting towards the end of the project. We suggest trainees may want to consider implementing more robust engagement strategies in their projects, for example (1) hosting teleconferences, video conferences, or face-to-face team meetings throughout the project to discuss topics such as how team members feel the study is progressing, how KUs feel about their contribution to the project and their role(s), and identifying potential sources of conflict; (2) and having a platform, such as a web-based forum, to facilitate discussion between all team members throughout the duration of the project. Such strategies may have prevented or minimized KU turnover in this project. In addition, having a larger group of KUs at the beginning of the study would have provided a sufficient number of KUs to carry on without having to replace them mid-way through. Therefore, students pursing an iKT thesis project with short timelines should consider having various engagement strategies in place prior to study onset, to minimize or prevent KU turnover.

Interestingly however, the fluid involvement of KUs leaving and joining the team during the project was less disruptive than we would have anticipated. As evidenced during the advisory panel meeting, all KUs (even those joining midway) voiced that they still identified with the research aims and found results applicable within their respective contexts. If anything, the advisory panel meeting sparked a bigger discussion on other risk factors that KUs believe to exist for these students, and the care and support in place (or lacking) for PS students with symptoms of psychosis. Furthermore, with the reality of changing research teams, there may be value in having team meetings, rather than bilateral one-on-one meetings (either face-to-face or virtually) throughout the project to allow for individuals to meet new team members and existing team members. There are many benefits of face-to-face meetings, including the development of trust and rapport among team members, allowing team members to learn about each other and their respective roles, and integrating information more effectively compared to virtual forms of communication.^19^ However, these types of team meetings require resources and thus need to be carefully planned when part of a graduate student project. In general, team meetings elicit deeper understanding of individual viewpoints,^15^ and fosters discussion of project priorities and progress.^13^ Based on our experience, virtual and asynchronous communication still allowed for interesting and thoughtful discussion and involvement of KUs who would otherwise be unavailable for a planned in-person meeting.

###  Evaluating Partnerships Within iKT Research

Evaluating partnerships is important when conducting iKT research as it helps identify potential partnership conflicts early, helps identify benefits and challenges of the partnership, and ensures KUs members feel comfortable and able to contribute to the partnership.^13^ There are a number of ways that evaluation can be built into a project, and can be formal or informal. Given the limited methodological guidance for iKT systematic reviews, formal evaluation to gather data on the method itself would strengthen the evidence base supporting the engagement of KUs in this type of research. Formal evaluation can also be used to systematically obtain information about the effectiveness of KU and researcher partnerships from a quality assurance lens, though the added workload for KUs must be acknowledged. On the other hand, informal processes might include (1) creating a timeline containing milestones for the iKT partnership so it can be easily tracked by all members; (2) gathering team member feedback after meetings to discuss how everyone thinks the project is going; and (3) having an open dialogue about the relationship and progress of the study.^13^ An informal evaluation strategy has the benefit of being flexible to accommodate the KUs schedules and other obligations. Regardless of the approach used, evaluation is vital to understand what KU engagement strategies work well, challenges that exist for maintaining relationships over the course of a study and understanding why turnover happens. Ultimately, by not evaluating our partnerships, we had little information on strategies and effectiveness for team member engagement, such as strengths, weaknesses, and suggestions for improvement. This information may have helped to explain some of the difficulties encountered during the project, especially KU turnover and reasons for it.

###  Benefits

Overall, KU team members ensured that project findings were relevant within their own clinical, community, and PS institution contexts by drafting study implications from their own perspectives. KUs played an active role in interpreting the findings and how study results may benefit clinicians, students, educators, and others involved with PS students with symptoms of psychosis. For example, they highlighted clinically relevant risk factors related to psychosis that were reported in the systematic review, such as depression and substance use, but also highlighted important risk factors that were inconclusive in the systematic review, such as anxiety, trauma, sleep dysfunction, and family history of mental illness,^12^ which they routinely flag when working with students. Overall, KUs emphasized the importance of clinicians performing routine risk assessments when working with PS students experiencing symptoms of psychosis. KUs also highlighted various interventions they utilize for PS students with symptoms of psychosis, such as systemic therapy. This intervention was identified in the systematic review, however it warrants further testing to determine its efficacy in assisting students with psychosis.^12^ KUs also highlighted that ‘system navigators’ is an intervention that has been implemented within PS institutions to assist students in navigating PS institution political structures, and academic and health issues. This intervention was not identified in our systematic review, therefore without KUs bringing up this potentially promising intervention, it would otherwise have gone unreported. Further details on systematic review results can be found elsewhere.^12^ These are a few examples to demonstrate the value of KU knowledge and how it can nuance and clarify synthesis findings – perhaps one of the most important take home messages for researchers using this approach.

KUs also provided ideas for dissemination of the research results. They suggested connecting with PS programs that provide mental health training for faculty and students, disseminating results through conferences, mental health forums, or online platforms, and soliciting a podcast host to speak about mental health issues in PS students informed by our study findings. Although dissemination is an on-going process, thus far, one article has been published to showcase systematic review results,^12^ and the PI presented the research study via a poster presentation at a conference recommended by a KU. The 6 KU team members who comprised the advisory panel have also been involved in writing a discussion article (in progress).

###  Adding to the Evidence Base

There were some notable similarities and differences between our study and the iKT-driven systematic review by Pollock and colleagues,^10^ and Hyde and colleagues.^11^ Like Pollock and colleagues, we formed a group of KUs with different backgrounds, experiences, and expertise, which enabled us to capture and incorporate diverse perspectives throughout the conduct of the study. Similar to Hyde and colleagues’ review,^11^ we involved KUs with designing the study protocol, interpreting the results, and planning the dissemination of study findings. iKT research is about redefining research roles and not expecting KUs to assume the role of traditional researchers or be experts in research methods – rather, we must take into account the distinct knowledge, expertise and experience each KU brings to the team.^20^ In our study, we had open discussions with each KU team member regarding how they wished to contribute to the project.

There are limitations with our study compared with the existing evidence base and were largely due to limited funding, and hosting one in-person group meeting, compared to previous work in which multiple meetings were held.^10,11^ Although we did not host multiple full-team in-person meetings, we were able to partner with and receive input from various KUs throughout the study. All KUs expressed their interest in the project, and were able to craft implications that are applicable within their respective contexts. In summary, graduate students considering iKT projects should carefully consider the potential importance and value of team meetings, and should take into account timelines for the project, available funding if in-person meetings are planned, and scope of the research project. In addition, we suggest formal (eg, vote counting) or in-formal (eg, open discussion) processes to achieve team consensus on the project’s objectives to better fit the scope of a master’s thesis project.

## Conclusion

 We engaged KUs as team members in various phases of a graduate student’s systematic review, from inviting KUs onto the research team to proposal development through to result interpretation and dissemination planning. KU team members changed during the study as some left the team and others joined it. Towards the end of the study, 6 KU team members joined the advisory panel and participated in an end of study meeting and directed the development of a dissemination plan. As a graduate student-led iKT systematic review, challenges included managing the scope of the project and maintaining the same KU team members throughout the project. We suggest graduate students pursuing iKT projects involve KUs as team members in the project as early as possible, have strategies in place to minimize or prevent team member turnover, host team meetings to foster on-going communication and to review project goals routinely, and develop a process for periodically checking in with team members to ensure engagement preferences are being met (ie, their roles and contributions). KUs helped interpret study findings using their unique expertise and offered implications for clinical practice, academic policy, education, future research, and dissemination ideas. Our experience in this project has been that a graduate student can conduct a thesis research study using an iKT approach and find the process feasible and rewarding.

## Acknowledgements

 We would also like to acknowledge knowledge users who were a part of the advisory panel, particularly Meriem Benlamri, Carmen Hust, April MacInnes, Crystal Morris, Fabien Provost, and Danielle J. Vigneault.

## Ethical issues

 This research was reviewed by the Health Sciences and Science Research Ethics Board at the University of Ottawa and was exempt from ethics review based on Article 2.1a of the Tri-Council Policy Statement-2.

## Competing interests

 Authors declare that they have no competing interests.

## Disclaimers

 The views expressed by authors in the submitted article are our own and are not an official position of the University of Ottawa.

## Authors’ contributions

 VS, AV, JDJ, and IDG were responsible for conception and design. VS was responsible for acquisition of data, data analysis and interpretation of data, and drafting of the manuscript. AV, JDJ, and IDG provided administrative, technical, material support, and supervision. VS, AV, JDJ, and IDG made critical revision of the manuscript for important intellectual content.

## Funding

 The study received no operational funding however VS received the Ontario Graduate Scholarship to support her Master of Science in Nursing degree. IDG is a recipient of a CIHR Foundation Grant (FDN #143237).

## Authors’ affiliations

^1^School of Nursing, Faculty of Health Sciences, University of Ottawa, Ottawa, ON, Canada. ^2^School of Epidemiology and Public Health, University of Ottawa, Ottawa, ON, Canada.
